# Stuttering after Intravenous Anesthesia: A Case Report

**DOI:** 10.31729/jnma.5343

**Published:** 2021-09-30

**Authors:** Suraj Lamichhane, Murari Raj Upreti, Yogesh Dhakal

**Affiliations:** 1 Department of Anesthesia, HAMS Hospital, Kathmandu, Nepal; 2 Department of Anesthesiology and Critical Care, B. P. Koirala Institute of Health Sciences, Dharan, Nepal

**Keywords:** *intravenous anesthesia*, *recovery*, *stuttering*

## Abstract

Stuttering is a form of speech disorder characterized by involuntary prolongation and repetition of sound, words, syllables or phrases as well as involuntary silent pauses or blocks. We report a case of a healthy twenty-six-year-old male patient without significant past history, who underwent short intravenous anesthesia for incision and drainage for perianal abscess. Postoperatively, the patient presented with prominent stuttering after six hours of surgery. To our knowledge, this is the first reported case of stuttering following short intravenous anesthesia without any airway manipulation. He was diagnosed with a functional speech disorder after excluding organic causes. His speech eventually normalized with six weeks of intensive speech therapy. This event posed a significant challenge for the surgical and anesthesia team to find the potential cause, to plan further management, and lead to two days prolongation of hospital stay.

## INTRODUCTION

Stuttering is a speech disorder that can cause disturbances in normal fluency and time patterning of speech. Stuttering can be developmental which is common under the age of five. Acquired onset is rare and can occur at any age and can occur after the head injury, stroke, progressive neurological disease, drugs, psychological or emotional trauma.^1^ Patient not able to speak or having speech disturbance after general anesthesia may be a horror for anesthesiologists as well as patients and relatives. Here we report a twenty-six-year-old male patient who has stuttering after undergoing incision and drainage for a perianal abscess under intravenous anesthesia.

## CASE REPORT

A twenty-six-year-old male patient presented to a tertiary care hospital in the surgery outpatient department (OPD) with the complaint of pain in the perianal region. He was diagnosed with a perianal abscess and planned for incision and drainage. The preoperative evaluation was done and there was no history of other medical illness and lab parameters were within normal limits. The patient was given the option of saddle block or intravenous anesthesia. The patient was anxious and preferred intravenous general anesthesia. Consent was taken and the patient was shifted to the operating room. Intravenous access was obtained and injection (Inj) cefazolin two gm and Inj. metronidazole 500mg was given as a prophylactic antibiotic and Inj. paracetamol one gm for pain management.

Routine standard monitoring was done which includes non-invasive blood pressure (NBP), heart rate (HR), pulse oximetry (SpO_2_), and electrocardiography (ECG). His baseline parameters were within the normal limit. Oxygen was given via facemask 6 l/min and breathing monitoring was done via capnography. The patient was induced with Inj. morphine four mg and Inj. propofol 150mg. The patient was kept in lithotomy position and incision and drainage was done. Anesthesia was maintained with Inj. propofol 100mg bolus and oxygen supplementation. The airway was maintained with chin lift and jaw thrust maneuver. The surgery lasted for 10 minutes and was uneventful with normal vitals intraoperatively.

The patient regained consciousness after five minutes of ending of surgery and was kept for observation in the recovery room. The patient was monitored for about an hour, during which he fully regained consciousness. He was able to speak clearly and recognize relatives and maintained a normal oxygen saturation level without oxygen. Then, he was shifted to ward. After about six-hour of surgery, the patient developed stuttering. Clinical examinations and investigations (Magnetic resonance imaging of head) didn't reveal any clear cause. The patient was discharged after two days of admission and asked to follow up for speech therapy. After six weeks of speech therapy, the patient had normal speech. Progress was steady throughout and he was regularly followed up via phone call.

## DISCUSSION

Speech disorders can be functional (with no known cause) or organic (developmental or acquired). Speech disorders can present in many forms like stuttering, dysphonia, aphonia. Stuttering is a form of speech disorder where there is marked disruption to the flow of the speech, prolongations in pauses, and repetition of words. Stuttering can be developmental stuttering which is common under the age of five. Acquired or late-onset which is rare and can occur at any age and can be neurogenic and psychogenic. In psychogenic stuttering, there is an associated emotional stressor or a traumatic event. There are no reported cases of stuttering after a patient received intravenous anesthesia without airway manipulation. So, we report a case of stuttering after intravenous anesthesia.

Narayanan et al. described a case of a forty-year-old female, without any previous illness, who developed mutism for one day after an uncomplicated general anesthetic for an emergency caesarean section.^2^ Differential diagnoses such as cerebral sinus venous thrombosis, reversible cerebral vasoconstriction syndromes, and postpartum angiopathy were excluded and any brain lesion was ruled out.^2^ Another case documented was a fifty-six-year-old female patient undergoing an umbilical hernia repair. She had a history of mutism after a recent GA that was attributed to the use of ketamine. After hernia repair also had mutism alongside weakness of limbs although no organic cause was found, later made a full recovery.^3^

Raphael and Schoenfield reported the case of a forty-four-year-old lady with a history of bipolar affective disorder, obesity, and a history of childhood sexual abuse who presented with a four-month history of stuttering two years after Roux-en-Y gastric bypass operation. The authors concluded that patients with a history of psychiatric illness and childhood sexual abuse might be predisposed to somatoform disorders.^4^ Another case reported was a female patient who underwent total intravenous anesthesia for femur fracture fixation and developed mutism for eleven days postoperatively but then made a full recovery by the eighteenth day. They concluded that propofol could have been the contributing factor to the speech disorder.^5^

Many drugs like antiepileptic, neuroleptics, antidepressants are also implicated in such cases but these drugs were not used in our case.^6^ In the reported cases for medication-induced stutter, it was noted that patients recovered from the speech impairment when the medication was ceased but our case made full recovery after six weeks of the first presentation. Awareness under general anesthesia may also lead to post-traumatic stress and stuttering and are common in cardiac surgery, cesarean sections, trauma patients in shock, and more when the neuromuscular blockade was used and significantly lower when no paralysis was involved.^7^ In our case report, our patient received no neuromuscular blockade and was a short procedure done under Inj. propofol and morphine only. However, depth of anaesthesia monitoring was not used on our patient either.

Our patient was anxious before the operation and asked for general anesthesia rather than a saddle block. The stress for surgery may be a reason for precipitating stuttering in this patient. However, drugs used like propofol as a causative agent for the event can't be ruled out. The management of stuttering may include pharmacological drugs like haloperidol, olanzapine, risperidone, and other dopamine blocking agents. Non-pharmacological treatments include affective, behavioral, cognitive aspects. Most established is speech therapy which is supported by literature and targets physiological centers of the brain. Our patient also had speech therapy and regained normal speech after six weeks.

Speech disorders like acquired stuttering could occur after general anesthesia which could be due to many potential factors like medications used, the stress of the surgery, psychosocial factors. Early recognition of abnormality and thorough neurological assessment and exclusion of possible organic causes are important. This event posed a significant challenge for the surgical and anesthetic team with regards to finding the potential causes and plan further management and lead to the prolongation of hospital stay. Preoperative counseling, allaying anxiety, and psycho-social support are important in minimizing such a rare complication.

## References

[1] Rajani C, NG Su C, Kaur N (2017). Prolonged neurologic dysfunction after a general anaesthetic.. Arch Clin Cases..

[2] Narayanan A, Tawfic QA, Kausalya R, Mohammed AK (2012). Speechless after general anaesthesia for caesarean section.. Middle East Journal of Anesthesiology..

[3] Gurunathan U, Iswariah H (2016). A case of mutism on emergence from general anesthesia.. J Anesth.

[4] Raphael DB, Schoenfeld FB (2006). Psychogenic stuttering following a gastric bypass operation: case report.. Jefferson J Psychiatry..

[5] Kati I, Demirel CB, Anlar O, Hüseyinoglu UA, Silay E, Elcicek K (2003). An unusual complication of total intravenous anesthesia: mutism.. Anesth Analg..

[6] Brady JP (1998). Drug-induced stuttering: a review of the literature.. J Clin Psychopharmacol..

[7] Pandit JJ, Andrade J, Bogod DG, Hitchman JB, Jonker WR, Lucas N (2014). 5th National Audit Project (NAP5) on accidental awareness during general anaesthesia: summary of main findings and risk factors.. Br J Anaesth..

